# Lutein Has a Positive Impact on Brain Health in Healthy Older Adults: A Systematic Review of Randomized Controlled Trials and Cohort Studies

**DOI:** 10.3390/nu13061746

**Published:** 2021-05-21

**Authors:** Ayano Yagi, Rui Nouchi, Laurie Butler, Ryuta Kawashima

**Affiliations:** 1 Department of Cognitive Health Science, Institute of Development, Aging and Cancer (IDAC), Tohoku University, Sendai 980-8575, Japan; ayano.yagi.e5@tohoku.ac.jp; 2 Smart Aging Research Center, Tohoku University, Seiryo-machi 4-1, Sendai 980-8575, Japan; ryuta@tohoku.ac.jp; 3 Faculty of Science and Engineering, Anglia Ruskin University, Cambridge CB1 1PT, UK; laurie.butler@aru.ac.uk; 4 Department of Functional Brain Imaging, Institute of Development, Aging and Cancer (IDAC), Tohoku University, Sendai 980-8575, Japan

**Keywords:** lutein, carotenoid, brain, MRI, VBM, systematic review

## Abstract

A previous systematic review revealed that lutein intake leads to improved cognitive function among older adults. However, the association between lutein intake and brain health remains unclear. Methods: We searched the Web of Science, PubMed, PsycInfo, and Cochrane Library for research papers. The criteria were (1) an intervention study using oral lutein intake or a cross-sectional study that examined lutein levels and the brain, (2) participants were older adults, and (3) brain activities or structures were measured using a brain imaging technique (magnetic resonance imaging (MRI) or electroencephalography (EEG)). Results: Seven studies using MRI (brain activities during rest, cognitive tasks, and brain structure) and two studies using EEG were included. We mainly focused on MRI studies. Three intervention studies using MRI indicated that 10 mg lutein intake over 12 months had a positive impact on healthy older adults’ brain activities during learning, resting-state connectivity, and gray matter volumes. Four cross-sectional studies using MRI suggested that lutein was positively associated with brain structure and neural efficiency during cognitive tasks. Conclusion: Although only nine studies that used similar datasets were reviewed, this systematic review indicates that lutein has beneficial effects on healthy older adults’ brain health.

## 1. Introduction

Vegetable and fruit intake is associated with cognitive function [1,2,3]. Several cohort studies have reported that vegetable and fruit intake enhances cognitive function among young and older adults [4,5,6,7]. In addition, the amount of vegetable and fruit intake affects brain function and structure [8]. Therefore, vegetable and fruit intake affect maintaining and improving cognition and the brain.

Lutein is one of the most abundant carotenoids in nature and the human diet. Carotenoids are naturally occurring pigments (red, yellow, or orange in color) and are found in vegetables and fruits [9,10]. Carotenoids have two chemical classes of carotenoids, hydrocarbon-based carotenes and xanthophylls, which also contain oxygen apart from the hydrocarbon component. Lutein is a xanthophyll, an antioxidant, and an anti-inflammatory agent in the human body [11]. Humans cannot synthesize carotenoids. Therefore, carotenoids are mainly derived from vegetables and fruits [12]. Lutein is mainly found in high quantities in green leafy vegetables and some fruits (e.g., spinach, kale, avocado, kiwi, etc.).

Meta-analyses and systematic reviews have revealed that regular lutein consumption reduces health problems such as cancer and eye disease (e.g., age-related macular degeneration) [13,14,15,16,17]. Moreover, a recent systematic review of randomized controlled trials (RCTs) indicated that lutein has a positive effect on cognition in young and older adults [18]. Notably, several neuroimaging studies have shown that lutein intake affects brain activity and brain structures [19,20,21,22]. However, to the best of our knowledge, no systematic review has investigated the association between lutein and brain functions and structures in humans. In this study, we conducted a systematic review that aimed to reveal the positive effects of lutein on the brain. In addition, this review focused on healthy older adults because of the importance of prevention and because the brains of healthy older adults have more plasticity than those of older adults with clinical problems.

## 2. Materials and Methods

### 2.1. Systematic Review Protocol and Registration

The systematic review protocol was designed using the International Prospective Register of Systematic Reviews (PROSPERO; registration no. CRD42020195957; https://www.crd.york.ac.uk/prospero/display_record.php?RecordID=195957, date of registration to PROSPERO, 29 July 2020). The protocol was based on the general principles of Preferred Reporting Items for Systematic Reviews and Meta-Analyses (PRISMA) [23] (Supplementary Table S1).

### 2.2. Search Strategy

We reviewed the literature on electronic databases such as PubMed, EMBASE, Web of Science, PsycInfo, and the Cochrane library, including the Cochrane Database of Systematic Reviews, Cochrane Central Register of Controlled Trials, Cochrane Methodology Register, Cochrane Collaboration Database of Abstracts of Reviews of Effects, Health Technology Assessment Database, and NHS Economic Evaluation Database. In the searches using each database, we used terms associated with the imaging of the human brain (Supplementary Table S2). We did not restrict studies based on the language or publication period.

### 2.3. Detail of Included Studies

#### 2.3.1. Types of Study

We included studies examining the effects of carotenoid intake using brain imaging in healthy older adults. The details of the search criteria were as follows: (1) An intervention study using oral lutein intake or a cross-sectional study that examined lutein levels and the brain, (2) participants were older adults, and (3) brain activities or structures were measured using a brain imaging technique (MRI or EEG; see Section 2.4).

#### 2.3.2. Participants

Participants in the systematic review were males and females aged 60 years or older. In studies comparing older people and young people, 1 group met the criteria. A study that included only individuals without cognitive impairment was included. We excluded participants with a current diagnosis or history of Alzheimer’s disease, head injury, depression, dementia, stroke, or other neurological disorders.

### 2.4. Main Outcomes

These studies involved the use of brain imaging data for analysis. Specifically, studies that measured brain activity (e.g., functional magnetic resonance imaging (fMRI) data at rest or during task performance or electroencephalography (EEG) data during task performance), structural MRI data (e.g., brain volume (gray/white matter), white matter integrity, and so on) such as fMRI data at rest or during task performance, and EEG data during task performance were eligible for this systematic review.

### 2.5. Data Extraction

The study titles and abstracts were screened using the systematic review search strategy. To determine if the studies met the inclusion criteria, 2 reviewers screened the information from the studies. Complete study reports for all potentially applicable studies were retrieved, and eligibility was assessed independently by 2 reviewers. The 2 reviewers discussed the eligibility of the studies until they reached agreements if they had different options.

## 3. Results

### 3.1. Search Results

**Figure 1:**
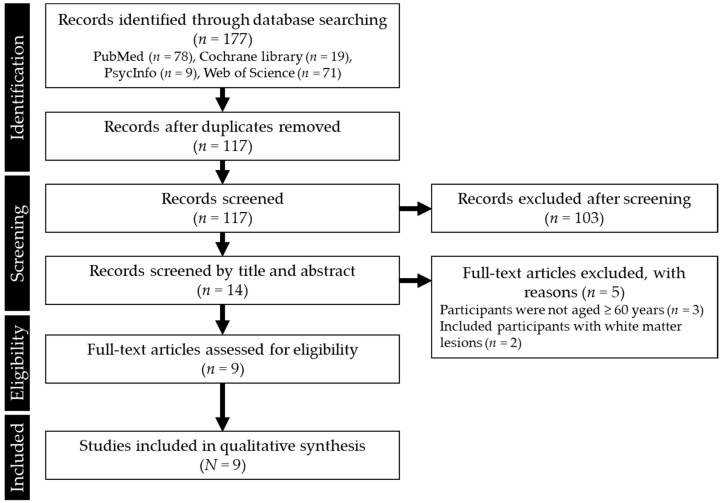
Study selection flow chart.

An electronic database search conducted between June and December 2020 resulted in 177 matching abstracts. Many papers reported on the components of carotenoids from the perspective of biochemistry. In addition, many studies on human subjects showed a relationship between carotenoids and cancer or obesity. Most of these papers included the word “t1” in their abstracts. Duplicate exclusions and screening of titles or abstracts identified 14 potentially eligible studies. Of these, five studies were excluded because participants were not aged ≥60 years (*n* = 3) or because they had white matter lesions (*n* = 2). The flowchart of study selection is shown in Figure 1.

### 3.2. Included Studies

Nine studies met the inclusion criteria for this systematic review (Table 1), of which four were intervention studies with a single-site, double-blind, RCT design [22,24,25,26]. Participants took either a supplement containing 10 mg lutein and 2 mg zeaxanthin or an inactive placebo of the same appearance. Participants took one tablet per day with their meal for 12 months. The other five studies examined the relationship between brain activity or brain structure and lutein concentration as measured by macular pigment optical density (MPOD) [21,27,28,29,30].

### 3.3. Participants

The sample sizes ranged from 43 to 92. The average age of the participants ranged from 20.58 to 72.51 years. All nine studies included older adults (aged ≥ 60 years), and two studies included young college students around 20 years old in the control group [27,30]. All included studies were carried out in the United States of America.

### 3.4. MPOD

Lutein and its isomer, zeaxanthin, accumulate in the inner layer of the macula of the retina and are called macular pigments. Macular pigment levels (quantified as optical density) can be measured noninvasively (MPOD). Macular pigment directly reflects the protection of the eye against high-energy light. MOPD was correlated with the concentration of macular carotenoids in the blood [31] and the brain [32]. The MPOD values for each study are presented in Table 2.

### 3.5. Outcomes and Imaging Methods

Seven studies used MRI [21,22,24,25,27,28,29] and two studies used EEG [26,30]. Concerning MRI, three studies measured brain activities during cognitive tasks such as memory encoding and retrieval [24], visual processing and decision-making [28], and verbal learning [29]. In addition, one MRI study used resting-state fMRI [22]. Three studies used T1 (T1-weighted image) and diffusion tensor imaging (DTI) to measure brain volume and white matter integrity [21,25,27]. Two studies used EEG measured brain activity during a visual attention task [26,30].

Three of seven MRI studies used RCT [22,24,25] and four of seven MRI studies used cross-sectional studies [21,27,28,29]. Three MRI studies using RCT reported greater activity in the dorsolateral prefrontal cortex (DLPFC) and anterior cingulate cortex (ACC) during memory tasks [24], increased functional integration during the resting state [22], and increased total gray matter volume and regional gray matter volume in the prefrontal regions [25] after the intervention when comparing the placebo and supplement groups. Two fMRI studies of four cross-sectional studies reported negative correlations between lutein levels and task-related brain activity [28,29]. Two other structural MRI studies reported higher white matter integrity in the cingulum [27] and greater regional gray matter volume in the parahippocampus gyrus [21]. One EEG study was a cross-sectional study that included both young and older adults [30], and another EEG study was an RCT among older adults [26]. EEG studies revealed that lutein level was associated with greater brain activity during visual attention tasks [26,30].

The outcomes and the main results of the included studies are shown in Table 3. In addition, Table S3 lists the brain coordinates reported in a previous study.

### 3.6. Quality Assessment

The methodological quality ratings of the studies were conducted based on previous studies [18,33]. The quality assessment scores ranged from 7 to 10 (see the Supplementary Table S4), indicating that all studies had adequate methodological quality.

## 4. Discussion

This was the first study to review the effects of lutein on the brain in healthy adults. The results consistently showed that lutein intake may positively affect brain activity during learning, as well as resting connectivity and gray matter volume in healthy older adults. In a cross-sectional study, lutein was also shown to positively affect brain structure and neural efficiency during a cognitive task in healthy older adults. Specifically, nine studies met the inclusion criteria for this systematic review [21,22,24,25,26,27,28,29,30]. All studies included older adults without dementia [21,22,24,25,26,27,28,29,30]. However, three studies reported that approximately 10% of the participants had a mild cognitive impairment (CDR = 0.5) [25,26,30]. Seven studies used MRI [21,22,24,25,27,28,29] and two studies used EEG [26,30]. Three of seven MRI studies used RCT [22,24,25] and four of seven MRI studies used a cross-sectional design [21,27,28,29]. We mainly discussed MRI studies because only two EEG studies were found.

Of the three RCT studies, one study measured brain activities during encoding and retrieval tasks [24], one study measured resting-state brain activities [22], and one study used structural MRI using T1-weighted images and DTI [25]. All studies shared a similar dataset. Participants consumed a pill that included 10 mg lutein and 2 mg zeaxanthin per day for 12 months. One fMRI study using a memory task reported that the lutein intake group showed greater activity in the left DLPFC and ACC during the encoding and retrieval after the 12 months intervention period [24] compared to the baseline, but the placebo group did not. The resting-state fMRI study [22] also revealed that the lutein intake group showed an increase in functional integration between default mode networks and other resting-state networks (e.g., executive control, auditory, and frontoparietal) after the intervention period, but the placebo group did not [22]. The structural MRI study did not find any significant changes in the white matter microstructure (e.g., FA, MD, RD, and AD). However, in the supplement group, older adults who had increased lutein levels during the intervention period showed small declines in total gray matter and prefrontal gray matter volume compared to older adults who were stable or had decreased lutein levels during the intervention period [25]. These results suggest that 10 mg lutein intake over 12 months may positively impact brain activity during learning, as well as resting-state connectivity and gray matter volumes in healthy older adults.

Of the cross-sectional studies, two studies measured brain activity during visual-spatial processing [28] and verbal learning [29]. Two studies used structural MRI with DTI [27] and T1-weighted images [21]. Three studies shared a similar dataset [27,28,29]. fMRI studies have consistently reported negative correlations between lutein levels and task-related brain activity [28,29]. For example, older adults with lower (higher) lutein levels showed increased (decreased) brain activities in the key brain region related to visual and spatial processing and decision-making during judgment of the line orientation task [28]. In addition, older adults with lower (higher) lutein levels needed greater (lower) brain activities in several brain regions, including the frontal, temporal, parietal, and occipital lobes, during verbal learning [29]. One structural MRI study revealed that lutein levels were positively associated with FA in the cingulum in older adults [27]. This means that older adults with higher lutein levels have higher white matter integrity than their counterparts. Another structural MRI study reported that the gray matter volume in the right parahippocampal gyrus was positively associated with lutein levels in the serum of healthy older adults [21]. Consequently, lutein has a positive effect on brain structure and neural efficiency during cognitive tasks.

It is important to consider the potential biological mechanisms underlying the effects of lutein on the human brain. Based on the previous hypothesis [18], we inferred the mechanisms by which lutein and its isomers affect brain activity and structure. Lutein and its isomer have antioxidant and anti-inflammatory effects in vivo [34] and traverse the blood-brain barrier [35]. In particular, lutein mainly exists in the frontal and visual brain areas and hippocampus [36,37]. The amount of lutein and its isomers in the prefrontal cortex are higher than those in other brain regions [36]. Reduction of food-intake-related oxidant levels leads to changes in brain activation [38,39]. The anti-inflammatory properties of lutein increase the production of brain-derived neurotrophic factor in the brain [40], which facilitates neural plasticity [41]. Lutein and its isomer selectively perform antioxidant and anti-inflammatory functions in the frontal cortex, hippocampus, and visual cortex because their concentrations in these brain regions are higher than those in other brain regions [36,37]. Therefore, the intake of lutein and its isomer has a positive impact on specific brain regions, such as the frontal and occipital lobes.

These results suggest that daily consumption of lutein may help prevent cognitive decline in aging [42]. However, in recent years, it has been pointed out that the average daily intake of lutein is below the recommended intake [43]. Lutein has been shown to be abundant in green leafy vegetables [44], so it would be better to consume them actively.

This systematic review has some limitations. Only seven MRI studies were included (three RCTs and four cross-sectional studies). However, they used different MRI methods (fMRI or structural MRI). Three of four fMRI studies measured brain activity during different cognitive tasks (memory encoding and retrieval, visuospatial tasks) [24,28,29], and one of four fMRI studies measured resting-state fMRI [22]. Whereas brain plasticity has been observed between several types of cognitive tasks, the reproducibility of the results is questionable because only a single paper has reported on each task. Two studies of three-structure MRI used T1-weighted images to analyze gray or white matter volumes [21,25]. Two studies of three-structure MRI used DTI to analyze FA, RD, MD, or AD [25,27]. Owing to the small number of studies and the wide range of MRI analysis methods, it would be difficult to perform a meta-analysis [45]. The second limitation is that six of the seven studies used a similar dataset [22,24,25,27,28,29]. This raises questions about the generality of the results. The fact that these studies used a dataset of experiments conducted by the same group also raises questions about the reproducibility of the study. In addition, all the studies were conducted in North America. It is important to investigate the beneficial effects of lutein on the brain in different countries and regions.

## 5. Conclusions

This novel systematic review investigated the effects of lutein on the brain. From three RCT studies and four cross-sectional studies, we found that lutein has beneficial effects on brain function and brain structure in older adults. In addition, 12 months of 10-mg lutein intake selectively affected brain activity and the total gray matter volume in the prefrontal cortex of older adults. In conclusion, this systematic review indicated that lutein has a positive impact on brain health in healthy older adults.

## Figures and Tables

**Table 1:** Studies characteristics.

Lead Author; Year; Country	Study Design; Duration	Sample Size (Female)	Age (Years) (Mean ± *SD*)	Health Status	Intervention (Timing or Method)	Control (Contents)	Imaging Method
Lindbergh; 2018; Georgia [24]	A single-site, double-blind RCT; 12 months	44 (26) P: 14 A: 30	P: 70.43 ± 5.43 A: 72.43 ± 6.48	Community-dwelling older adults; good overall health	Consumed one pill per day with a meal (L: 10 mg + Z: 2 mg/placebo)	Placebo (*n*/R)	fMRI
Lindbergh; 2020; Georgia [22]	A single-site, double-blind RCT; 12 months	48 (28) P: 14 A: 34	P: 70.43 ± 5.43 A: 73.06 ± 6.48	Community-dwelling older adults; good overall health; older adults without dementia	Consumed pills per day with a meal (L: 10 mg + Z: 2 mg/placebo)	Placebo (*n*/R)	fMRI
Mewborn; 2019; Georgia [25]	A single-site, double-blind RCT; 12 months	47 (27) P: 14 A: 33	P: 72.4 ± 6.27 A: 70.4 ± 5.43	Community-dwelling older adults; good overall health; older adults without dementia (CDR = 0.5, P: 05; A: 12.1%)	Took one tablet from the bottle daily with a meal (L: 10 mg + Z: 2 mg/placebo)	Placebo (*n*/R)	sMRI
Ceravolo; 2019; Georgia [26]	A single-site, double-blind RCT; 12 months	50 P: 15 (4) A: 35 (18)	P: 72.51 ± 6.24 A: 70.87 ± 5.50	Community-dwelling older adults; good overall health; older adults without dementia (included CDR = 0.5)	Received either 10 mg of L + 2 mg of Z per day	Placebo (*n*/R)	EEG
Mewborn; 2018a; Georgia [27]	Cross-sectional study as part of a larger RCT; one-shot	O: 54 (31) Y: 38 (17)	O: 71.87 ± 6.05 Y: 20.58 ± 2.02	Healthy men and women; older adults without dementia	No intervention	--	sMRI
Mewborn; 2018b; Georgia [28]	Cross-sectional study as part of a larger RCT; one-shot	51 (30)	71.75 ± 6.16	Community-dwelling older adults; good overall health; older adults without dementia	No intervention	--	fMRI
Lindbergh; 2017; Georgia [29]	Cross-sectional study; one-shot	43 (25)	71.55 ± 5.84	Community-dwelling older adults; good overall health	No intervention	--	fMRI
Zamroziewicz; 2016; Illinois [21]	Cross-sectional study; one-shot	76 (50)	69 ± 3	Healthy men and women; older adults without dementia	No intervention	--	sMRI
Oliver; 2019; Georgia [30]	Case-control design study as part of a larger cross-sectional study; one-shot	O: 42 (26) Y: 43 (20)	O: 72.36 ± 6.58 Y: 20.79 ± 2.16	Older adults without dementia (CDR = 0.5, Y: N/A; O: 9.5%)	No intervention	The stimuli presented and the task Instructions were controlled	EEG

RCT: Randomized control trial; O: Older adults; Y: Young adults; P: Placebo control group; A: Active intervention group; CDR: Clinical dementia rating; L: Lutein; Z: Zeaxanthin; *n*/R: Not reported; fMRI: Functional magnetic resonance imaging; sMRI: Structural magnetic resonance imaging; EEG: Electroencephalography.

**Table 2:** The MPOD for each study.

Lead Author; Year	Group	Subgroup	MPOD		Serum Nutrients Lutein		
			Baseline	Post	(μmol/mL)	*p* Values	Effect Size
			*M* (*SD*)	*M* (*SD*)	*M* (*SD*)	(*t*-test)	(Cohen’s *d*)
Lindbergh; 2018 [24]	Placebo		0.44 (0.14)	0.44 (0.19)		0.961	0.03
	Supplement		0.54 (0.19)	8.80 (2.16)		0.016	0.95
Lindbergh; 2020 [22]	Placebo		0.44 (0.14)	0.44 (0.19)		0.961	0.03
	Supplement		0.50 (0.21)	0.57 (0.23)		0.008	0.98
Mewborn; 2019 [25]	Placebo	Responder	0.45 (0.20)	0.69 (0.23)			
		Non-responder	0.57 (0.17)	0.51 (0.17)			
	Supplement	Responder	0.39 (0.16)	0.594 (0.16)			
		Non-responder	0.50 (0.99)	0.37 (0.18)			
Ceravolo; 2019 [26]	Placebo		0.47 (0.17)	-		Non-significant	
	Supplement		0.52 (0.18)	0.58 (0.23)		<0.03	
Mewborn; 2018a [27]	Younger adults		0.43 (0.16)				
	Older adults		0.50 (0.17)				
Mewborn; 2018b [28]			0.50 (0.18)				
Lindbergh; 2017 [29]			0.51 (0.18)				
Zamroziewicz; 2016 [21]					454 (275)		
Oliver; 2019 [30]	Younger adults		0.43 (0.17)				
	Older adults		0.50 (0.19)				

MPOD: Macular pigment optical density; *M*: Mean; *SD*: Standard deviation.

**Table 3:** Description of correlation between lutein or carotenoid and brain regions.

Lead Author; Year	Imaging Method	Results
Lindbergh; 2018 [24]	fMRI while participants were engaged in a verbal learning task	An enhanced BOLD signal in select ROIs, including left dorsolateral prefrontal cortex and anterior cingulate cortex
Lindbergh; 2020 [22]	Resting-state fMRI	An enhanced correlation of default mode network to other functional networks
Mewborn; 2019 [25]	MRI (T1-weighted, DTI)	Did not appear to influence age-related reductions for frontal and medial-temporal gray and white matter (exploratory analyses: individuals who showed greater increases in MPOD had less reduction in global and prefrontal gray matter volume than supplement “non-responders”)
Ceravolo; 2019 [26]	EEG while participants looked at stimuli to elicit the steady-state visual evoked potentials	Supplementation with L and Z changed both the power at the drive frequencies and the signal-to-noise ratio at those frequencies changed
Mewborn; 2018a [27]	MRI (DTI)	Higher L and Z concentrations predicted better white matter integrity in older adults
Mewborn; 2018b [28]	fMRI while participants were engaged in a judgment of line orientation task	Higher concentrations of L and Z decreased BOLD signal during task performance in key areas related to visual-spatial perception, decision-making, processing, and motor coordination
Lindbergh; 2017 [29]	fMRI while participants were engaged in a verbal learning task	MPOD was associated with activity in regions involved in language processing/serum L and Z predicted activity in regions involved in somatosensory functions
Zamroziewicz; 2016 [21]	MRI (T1-weighted)	Gray matter thickness only in the right parahippocampal cortex mediated the relationship between serum lutein and crystallized intelligence
Oliver; 2019 [30]	EEG while participants were engaged in an attentionally taxing task	MPOD covaried with visual attention

Note: L: Lutein; Z: Zeaxanthin; fMRI: Functional magnetic resonance imaging; EEG: Electroencephalography; DTI: Diffusion tensor imaging; MPOD: Macular pigment optical density; BOLD: Blood oxygenation level dependence; ROI: Region of interest.

## Data Availability

Not applicable.

## Supplementary Materials

The following are available online at https://www.mdpi.com/article/10.3390/nu13061746/s1, Table S1: PRISMA 2009 Checklist, Table S2: Search terms and strategy, Table S3: The brain coordinates, Table S4: Quality assessment scores of intervention studies included in the list.
