# Supplementary material for: Lutein Has a Positive Impact on Brain Health in Healthy Older Adults: A Systematic Review of Randomized Controlled Trials and Cohort Studies
Source: Nutrients. 2021 May 21;13(6):1746. doi: 10.3390/nu13061746 (PMC8223987; doi:10.3390/nu13061746)
Supplement: Supplementary file 1 [file nutrients-13-01746-s001.zip › nutrients-1191666-supplementary/supplementary/TS2_search.pdf]

Search terms

PubMed& other databases (EMBASE, Web of Science, PsycINFO)

We use “Mesh” filter which can only use at PubMed in the below search terms, however, the search terms will be adapted for use with various bibliographic databases in combination with database specific filters, where available when we search other databases.

#1	"lutein"[Title/Abstract] OR "carotenoid"[Title/Abstract]
#2	"magnetic resonance imaging"[Title/Abstract] OR "mri"[Title/Abstract] OR ("electroencephalography"[Title/Abstract] OR "electroencephalography"[Title/Abstract] OR "eeg"[Title/Abstract]) OR "gray matter"[Title/Abstract] OR "grey matter"[Title/Abstract] OR "white matter"[Title/Abstract] OR "VBM"[Title/Abstract] OR "T1"[Title/Abstract] OR "DTI"[Title/Abstract] OR "resting state"[Title/Abstract] OR "default mode"[Title/Abstract]
#3	NOT ("animals"[MeSH Terms:noexp] OR animal[Title/Abstract]) NOT ("rats"[MeSH Terms] OR "rats"[Title/Abstract] OR "rat"[Title/Abstract]) NOT ("mice"[MeSH Terms] OR "mice"[Title/Abstract] OR "mouse"[Title/Abstract])
#4	#1 AND #2 AND #3

Cochrane library

#1	(lutein):ti,ab,kw
#2	(carotenoid):ti,ab,kw
#3	(mri):ti,ab,kw
#4	(magnetic resonance imaging):ti,ab,kw
#5	(electroencephalography):ti,ab,kw
#6	(electroencephalography):ti,ab,kw
#7	(eeg):ti,ab,kw
#8	(gray matter):ti,ab,kw
#9	(grey matter):ti,ab,kw
#10	(white matter):ti,ab,kw
#11	(VBM):ti,ab,kw
#12	(T1):ti,ab,kw
#13	(DTI):ti,ab,kw
#14	(resting state):ti,ab,kw
#15	(default mode):ti,ab,kw
#16	animal
#17	(rats):ti,ab,kw
#18	MeSH descriptor: [Rats] explode all trees
#19	MeSH descriptor: [Animation] explode all trees
#20	(rat):ti,ab,kw
#21	MeSH descriptor: [Mice] explode all trees
#22	(mice):ti,ab,kw
#23	(mouse):ti,ab,kw
#24	(#1 or #2) and (#3 or #4 or #5 or #6 or #7 or #8 or #9 or #10 or #11 or #12 or #13 or #14 or #15) not (#16 or #17 or #18 or #19 or #20 or #21 or #22 or #23)
