# Supplementary material for: Lutein Has a Positive Impact on Brain Health in Healthy Older Adults: A Systematic Review of Randomized Controlled Trials and Cohort Studies
Source: Nutrients. 2021 May 21;13(6):1746. doi: 10.3390/nu13061746 (PMC8223987; doi:10.3390/nu13061746)
Supplement: Supplementary file 1 [file nutrients-13-01746-s001.zip › nutrients-1191666-supplementary/supplementary/TS3_the brain coordinates.pdf]

Table S3. The brain coordinates.

**Lead author; year**	**Target relationships**	**Region**	**Voxels**	***p***	***x***	***y***	***z***	**Extent**	***T***	***Z***	**Effect Size (*r*)**
Mewborn; 2018a	Relation of MPOD and Serum L & Z to ROI diffusivity	FA—Serum									
		L Cingulum	8	.019	−7	11	27		3.79		.465
		RD—Serum									
		L Cingulum	7	.033	−6	9	29		3.29		.415
		L Cingulum	2	.041	−7	13	26		3.12		.397
		AD—MPOD									
		L Uncinate fasciculus	4	.040	−36	−1	−16		3.53		.439
	Relation of MPOD and Serum L & Z to exploratory whole-brain diffusivity	FA—MPOD									
		R Genu of the corpus callosum	1	.002	14	25	−7		3.22		.408
		L Fornix	1	.010	−24	−30	2		2.62		.342
		R Posterior corona radiata	1	.001	25	−36	25		3.55		.442
		FA—Serum									
		L Posterior corona radiata	4	.003	−27	−62	20		3.02		.385
		L Superior corona radiata	1	.001	−28	−14	34		4.00		.485
		RD—MPOD									
		L Cingulum (hippocampus)	5	.008	−24	−22	−22		2.89		.372
		R Genu of the corpus callosum	1	.004	14	25	−7		2.84		.366
		R Posterior corona radiata	1	.009	25	−35	36		2.43		.319
		R Superior corona radiata	1	.008	22	2	31		2.44		.321
		RD—Serum									
		R Posterior thalamic radiation	1	.010	38	−54	0		2.31		.305
		L Superior corona radiata	1	.002	−28	−14	34		3.33		.419
		AD—MPOD									
		L Uncinate fasciculus	8	.001	−36	−1	−16		3.53		.440
		L Uncinate fasciculus	1	.006	−35	−1	−21		2.64		.345
		L Uncinate fasciculus	1	.009	−37	−4	−17		1.95		.261

		AD—Serum								
		L Cingulum (hippocampus)	3	.009	−24	−17	−26		2.50	.328
		L Cingulum (hippocampus)	1	.008	−26	−19	−25		2.96	.380
		R Uncinate fasciculus	1	.003	32	0	−20		3.12	.397
		L Corticospinal tract	2	.008	−5	−19	−22		2.10	.280
Mewborn ; 2018b	Relationship between MPOD and brain activation	R superior lateral occipital cortex			54	−70	16	19	3.08	.403
					56	−66	16	*	2.90	.383
		R inferior lateral occipital cortex			56	−68	12	*	2.94	.387
		R middle frontal gyrus			48	18	30	59	3.03	.397
		R frontal pole			0	60	16	31	2.93	.386
		L cingulate gyrus			−2	4	24	30	2.85	.377
		R cingulate gyrus			4	2	30	*	2.56	.343
		R angular gyrus			60	−50	38	15	2.84	.376
					62	−50	34	*	2.81	.373
		R precentral gyrus			62	12	8	9	2.82	.374
		R superior frontal gyrus			6	56	25	10	2.62	.351
	Relationship between Serum L & Z and brain activation	R precentral gyrus			16	−18	48	62	3.75	.492
		L middle temporal gyrus			−56	−8	−12	51	3.55	.472
		L superior parietal lobule			−36	−42	50	199	3.46	.462
					−28	−56	58	*	3.29	.444
		L superior lateral occipital cortex			−20	−74	40	61	3.42	.458
					−16	−88	36	12	2.73	.381
		R temporal fusiform cortex			36	−30	−16	33	3.36	.452
		L precentral gyrus			−20	−40	44	68	3.32	.448
		R superior lateral occipital cortex			26	−84	30	67	3.21	.436
					24	−62	56	51	3.04	.417
		L superior frontal gyrus			−22	30	54	28	3.16	.430
		L posterior superior temporal gyrus			−60	−32	4	49	2.92	.403
		L planum temporale			−62	−22	6	*	2.70	.377
		L superior temporal gyrus			−52	−38	2	*	2.56	.360

		L occipital pole	−28	−96	6	9	2.99		.411
		L temporal fusiform cortex	44	−16	−16	9	2.95		.406
		R thalamus	24	−22	4	36	2.93		.404
		L planum polare	−36	−8	−10	19	2.87		.397
		R posterior supramarginal gyrus	46	−38	10	21	2.58		.362
		R posterior superior temporal gyrus	54	−36	8	*	2.87		.397
		L Heschl's gyrus	−46	−22	2	12	2.83		.392
		L parahippocampal gyrus	−32	−36	−18	22	2.83		.392
		R postcentral gyrus	6	−40	62	22	2.66		.372
		L central opercular cortex	−48	6	2	11	2.75		.383
		R anterior middle temporal gyrus	58	−2	−22	19	2.70		.377
		R anterior superior temporal gyrus	50	−2	−16	*	2.62		.367
		L temporal pole	−52	6	−18	10	2.67		.373
		R lingual gyrus	18	−60	−16	8	2.61		.366
Lindbergh; 2017	Relationship of Serum L & Z to brain activation during encoding	MPOD							
		L insular cortex	-40	10	-14	99		3.03	.45
		L insular cortex	-42	0	-14	*		2.94	.44
		R middle temporal gurus	62	-58	-10	10		2.75	.41
		L cerebellum	-10	-76	2	11		2.52	.38
		L supramarginal gyrus	-64	-34	-22	3		2.44	.37
		Serum			26				
		L lateral occipital cortex	-24	-74	38	45		2.96	.44
		L postcentral gyrus	-20	-44	66	31		2.90	.43
		L parietal operculum cortex	-48	-30	24	39		2.90	.43
		L precentral gyrus	-58	0	32	5		2.76	.41
		R lateral occipital cortex	36	-68	50	17		2.60	.39
		R lateral occipital cortex	26	-78	28	7		2.48	.37
	Relationship of Serum L & Z to brain	MPOD							
		L inferior frontal gyrus	-42	8	24	48		3.10	.46
		L cerebellum	-10	-74	-22	24		2.96	.44
		L occipital pole	-12	-102	-2	9		2.78	.41

activation during recall	L planum polrare	-46	-4	-6	8	2.56	.38
	L insular cortex	-38	-4	-12	15	2.53	.38
	R middle frontal gyrus	46	34	18	7	2.47	.37
	R occipital pole	16	-96	12	2	2.40	.36
	Serum						
	L central opercular cortex	-48	-4	10	21	3.36	.49
	R lateral occipital cortex	22	-68	58	9	2.56	.38
	L central opercular cortex	-58	2	2	7	2.48	.37
	L superior parietal lobule	-38	-42	60	4	2.45	.37

FA: fractional anisotropy. RD: radial diffusivity. AD: axial diffusivity. R: right. L: left. ROI: region of interest. Serum L: lutein. Z: zeaxanthin. MPOD: macular pigment optical density. *cluster overlaps with the preceding row.
