# Supplementary material for: Lutein Has a Positive Impact on Brain Health in Healthy Older Adults: A Systematic Review of Randomized Controlled Trials and Cohort Studies
Source: Nutrients. 2021 May 21;13(6):1746. doi: 10.3390/nu13061746 (PMC8223987; doi:10.3390/nu13061746)
Supplement: Supplementary file 1 [file nutrients-13-01746-s001.zip › nutrients-1191666-supplementary/supplementary/TS4_quality assessment.pdf]

Table S4. Quality assessment scores of intervention studies included in the list.

Lead author; year	Q1	Q2	Q3	Q4	Q5	Q6	Q7	Q8	Q9	Q10	Q11	Q12
Lindbergh; 2018	Y	Y	?	Y	Y	Y	Y	?	Y	N	Y	Y
Lindbergh; 2020	Y	Y	Y	Y	Y	Y	Y	?	Y	N	Y	Y
Mewborn; 2019	Y	Y	?	Y	Y	Y	Y	?	Y	N	Y	Y
Ceravolo; 2019	Y	Y	Y	Y	?	?	Y	?	?	Y	Y	?

Q1: Random allocation. Q2: Concealed allocation. Q3: Similar baselines among the groups. Q4: Eligibility specified. Q5: Blinded assessor outcomes. Q6: Blinded care provider. Q7: Blinded patient. Q8: Intention-to-treat analysis. Q9: Details of allocation method. Q10: Adequate descriptions of each group. Q11: Statistical comparison between groups. Q12: Dropout report. Y: Yes, the study met the criteria for the question. N: No; the study did not meet the criteria for the question. ?: No information or study was the case with the question.
